# Comparison of Mask Oxygen Therapy and High-Flow Oxygen Therapy after Cardiopulmonary Bypass in Obese Patients

**DOI:** 10.1155/2018/1039635

**Published:** 2018-01-28

**Authors:** Mazlum Sahin, Helin El, Ibrahim Akkoç

**Affiliations:** ^1^Department of Cardiovascular Surgery, Haseki Teaching and Research Hospital, Istanbul, Turkey; ^2^Department of Cardiovascular Surgery, Sisli Teaching and Research Hospital, Istanbul, Turkey; ^3^Department of Anesthesia and Reanimation, Haseki Teaching and Research Hospital, Istanbul, Turkey

## Abstract

**Background:**

To clarify the efficiency of mask O_2_ and high-flow O_2_ (HFO) treatments following cardiopulmonary bypass (CPB) in obese patients.

**Methods:**

During follow-up, oxygenization parameters including arterial pressure of oxygen (PaO_2_), peripheral oxygen saturation (SpO_2_), and arterial partial pressure of carbon dioxide (PaCO_2_) and physical examination parameters including respiratory rate, heart rate, and arterial pressure were recorded respectively. Presence of atelectasia and dyspnea was noted. Also, comfort scores of patients were evaluated.

**Results:**

Mean duration of hospital stay was 6.9 ± 1.1 days in the mask O_2_ group, whereas the duration was significantly shorter (6.5 ± 0.7 days) in the HFO group (*p*=0.034). The PaO_2_ values and SpO_2_ values were significantly higher, and PaCO_2_ values were significantly lower in patients who received HFO after 4th, 12th, 24th, 36th, and 48th hours. In postoperative course, HFO leads patients to achieve better postoperative FVC (*p* < 0.001). Also, dyspnea scores and comfort scores were significantly better in patients who received HFO in both postoperative day 1 and day 2 (*p* < 0.001, *p* < 0.001 and *p*=0.002, *p*=0.001, resp.).

**Conclusion:**

Our study demonstrated that HFO following CPB in obese patients improved postoperative PaO_2_, SpO_2_, and PaCO_2_ values and decreased the atelectasis score, reintubation, and mortality rates when compared with mask O_2_.

## 1. Introduction

According to World Health Organization, obesity is defined as excessive and abnormal fat accumulation that creates risk for health, and a person with a body mass index (BMI) greater than or equal to 30 kg/m^2^ is considered obese [1]. Incidence of sedentary lifestyle with high-fat diet intake and comorbidities including metabolic syndrome, diabetes mellitus, and hypertension are common among obese patients. Also, it is well known that obesity and obesity-related disorders are risk factors for severe coronary artery diseases which may require cardiopulmonary bypass (CPB) [2]. Additionally, obese patients are more vulnerable to pulmonary complications following CPB due to decreased total lung capacity, functional residual capacity, forced vital capacity, and expiratory reserve volume. Moreover, anesthetic agents and sedatives aggravate the respiratory instability in obese patients [3].

Previous studies had demonstrated that severe hypoxemia, hypercapnia, and prolonged apnea periods deteriorate healing after CPB. To avoid these undesirable conditions, some authors recommended oxygen therapy which had a key role in improving respiratory functions and patient comfort by decreasing desaturation episodes and reintubation rates after cardiac surgery [4, 5]. In a prospective randomized study, Zhu et al. showed significant reduction of the reintubation rate after cardiac surgery with mask O_2_ [5]. In contrast, Stéphan et al. reported insufficiency of mask O_2_ therapy for oxygen treatment following cardiac surgery-supported high-flow O_2_ (HFO) treatment [6]. Therefore, the most appropriate method of oxygen therapy is still under investigation, especially in obese patients who are at high risk for respiratory problems following CPB.

Although previous studies investigated the role of mask O_2_ and HFO treatments following CPB, none of these studies have compared these two different treatment modalities in patients with BMI more than 30 kg/m^2^. In this study, we, for the first time, aimed to clarify the importance of mask O_2_ and HFO treatments following CPB in obese patients.

## 2. Materials and Methods

In this prospective randomized study, charts of patients who underwent CABG in a tertiary academic center between January 2015 and January 2017 were analyzed. Ethical approval for the study was obtained from the Haseki Teaching and Research Hospital's Regional Ethical Committee, with study ID number 499. Patients with BMI > 30 kg/m^2^ were enrolled in the study. Randomization was done by a computer-based random number-sequencing program. Exclusion criteria were hemodynamic instability, patients younger than 18 years of age, and patients with tracheostomy, obstructive sleep apnea, and active pulmonary disease. Also, patients with low cardiac output and operations who were held under emergency conditions were excluded from the study. Written consent was obtained from patients and/or relatives.

### 2.1. Study protocol

In Cardiovascular Surgery Intensive Care Unit (ICU), every patient, who underwent CABG, was followed up by a well-trained ICU nurse and a cardiovascular surgeon in the postoperative period. Hemodynamically stable patients, who had a sufficient oxygenation (SpO_2_ (peripheral oxygen saturation) > 92, FiO_2_ (fraction of inspired oxygen) ≤ 0.4, PEEP (positive end-expiratory pressure) ≤ 8 mmHg, and PaO_2_ (arterial pressure of oxygen)/FiO_2_ ≥ 150), were weaned by the spontaneous breathing trial (SBT) with low-level pressure support or oxygen T-piece for 90–120 minutes. Endotracheal extubation was performed in patients who have tolerated the SBT.

After endotracheal extubation, patients were randomly divided into two groups. In patients (*n* = 50) who received HFO treatment, high-flow humidified oxygen (44 mm/H_2_O/L and 37°C) was released through a nasal cannula continuously with Optiflow (Vapotherm, New Hampshire, USA). The preliminary flow rate was 25–40 L/min, and the initial FiO_2_ was 50% to maintain SaO_2_ > 93. In the other group (*n* = 50 patients) who underwent oxygen therapy, oxygen was delivered from a simple face mask (Orya Medikal, Istanbul, Turkey) with a flow of 2–4 liters in a minute to maintain SpO_2_ > 93%. Active respiratory physiotherapy was performed for all patients during the postoperative period. The patients were encouraged for early mobilization.

### 2.2. Atelectasis Scoring

Presence of atelectasis was evaluated by a postoperative chest X-ray and classified according to the radiological atelectasis score system (RAS) [7]. The RAS is divided into five categories (0: clear lung fields, 1: plate-like atelectasis or slight infiltration, 2: partial atelectasis, 3: lobar atelectasis, and 4: bilateral atelectasis), and chest X-rays were evaluated by a single radiologist who was blinded to mask O_2_ and HFO outcomes.

After the surgery, respiratory parameters and physical examination findings were recorded in 4th, 12th, 24th, 36th, and 48th hours, respectively. Patients' comfort and relief was evaluated once per day (1, very poor; 2, poor; 3, sufficient; 4, good; and 5, very good). Also, patients were assessed about the effectiveness of treatment once per day (2, marked improvement; 1, slight improvement; 0, no change; −1, slight deterioration; and −2, marked deterioration) [6].

Discontinuation of mask O_2_ or HFO therapy due to the side effects, requirement of additional treatment, or necessity of reintubation for mechanical ventilation was accepted as failure of the current treatment. Reintubation criteria were cardiovascular instability, respiratory arrest or respiratory acidosis (pH < 7.30 and PaCO_2_ ≥ 50 mmHg), encephalopathy, and clinical findings of exhaustion and refractory hypoxemia (arterial oxygen saturation < 88% with FiO_2_ = 100%). Reintubation was performed according to the physician's decision, if required. The length of ICU and hospital stay was recorded. Lastly, respiratory complications, extrapulmonary complications, and presence of mortality were documented.

During statistical analyses, values were evaluated as numbers, means, percentages, and intervals. Numbers and percentages were compared using the chi-square test. Before the comparison of means of values, the values were evaluated for homogeneity. Homogeneously distributed values were compared using Student's *t*-test, and heterogeneously distributed values were compared using the Mann–Whitney *U* test.

## 3. Results

**Figure 1:**
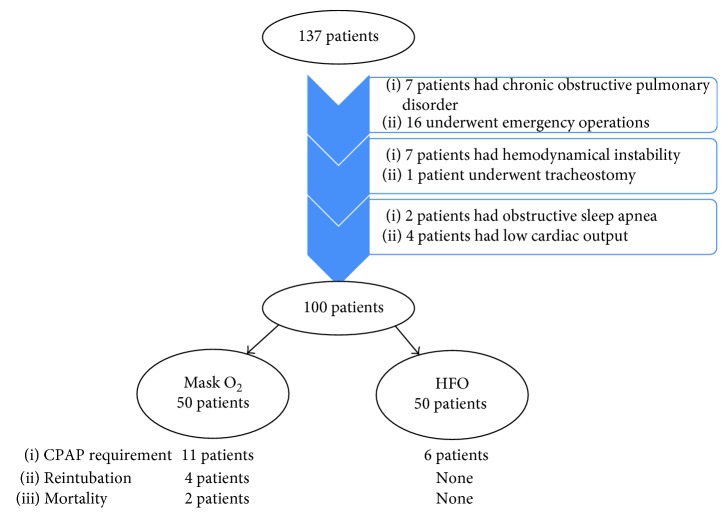
Flow chart scheme of the study.

During the study period, 137 patients underwent the CABG procedure, and 100 patients were enrolled in the study. Other 37 patients were excluded from the study according to study exclusion criteria (Figure 1). These patients were divided into two groups; 50 patients were treated with mask O_2_, and 50 patients were treated with HFO. The mean age was 61.3 ± 8.5 in patients who received mask O_2_ and 62.0 ± 6.7 in patients who received HFO (*p*=0.660). Sex, BMI, and history of smoking were similar between groups (*p*=1.000, *p*=0.259, and*p*=0.842, resp.). The operative parameters in terms of duration of surgery, myocardial ischemia period, and extubation time were comparable between patients who received mask O_2_ and HFO (*p*=0.709, *p*=0.740, and*p*=0.529, resp.). Preoperative and operative parameters are summarized in Table 1.

Among the patients who have received mask O_2_, 11 patients required continuous positive airway pressure (CPAP) and four patients required reintubation due to the increased arterial carbon dioxide value and mental deterioration despite extensive pulmonary rehabilitation maneuvers. In patients who have received HFO, CPAP was required in six patients; however, none of these patients required reintubation (*p*=0.187and*p* < 0.001). The duration of ICU stay was 2.8 ± 1.7 days in patients with mask O_2_ and 2.4 ± 0.5 days in patients with HFO (*p*=0.130). The duration of hospital stay was 6.9 ± 1.1 days in the mask O_2_ group, whereas it was significantly shorter (6.5 ± 0.7 days) in the HFO group (*p*=0.034). Moreover, the atrial fibrillation rate and mortality rate were significantly lower in patients who were treated with HFO (*p* < 0.001and*p* < 0.001, resp.). Death was observed in only two patients who have received mask O_2_ (Table 2).

The respiratory parameters PaO_2_ value and SpO_2_ value were significantly higher and PaCO_2_ value was significantly lower in patients who have received HFO. The PaO_2_ value was 104.3 ± 5.6, 96.2 ± 7.4, 96.6 ± 6.7, 97.1 ± 6.3, and 99.4 ± 7.1 after 4th, 12th, 24th, 36th, and 48th hours in patients with mask O_2_ and 112.3 ± 8.8, 106.9 ± 7.5, 100.0 ±4.5, 104.9 ± 5.9, and 106.0 ± 6.9 after 4th, 12th, 24th, 36th, and 48th hours in patients with HFO (*p* < 0.001, *p* < 0.001, *p*=0.004, *p* < 0.001, and*p* < 0.001, resp.). Similarly, SpO_2_ values were 98.0 ± 0.7, 97.5 ± 1.1, 97.4 ± 4.3, 97.5 ± 1.2, and 97.5 ± 1.2 after 4th, 12th, 24th, 36th, and 48th hours in patients with HFO. SpO_2_ values were statistically better in patients who have received HFO when compared with the mask O_2_ group (*p* < 0.001, *p* < 0.001, *p*=0.001, *p* < 0.001, and*p* < 0.001, resp.). In contrast, patients with mask O_2_ faced with worse PaCO_2_ values (43.1 ± 2.8 versus 41.2 ± 2.1, 43.7 ± 1.9 versus 41.2 ± 2.1, 43.5 ± 1.8 versus 40.7 ± 2.2, 43.3 ± 1.6 versus 39.3 ± 2.8, and 42.3 ± 2.2 versus 37.9 ± 2.6 after 4th, 12th, 24th, 36th, and 48th hours, resp.). The mean heart rate was significantly lower in the HFO group in 4th, 12th, 24th, and 36th hours after CPB (*p* < 0.001, *p*=0.009, *p*=0.001, and*p* < 0.001, resp.). In addition, patients who have received HFO had higher blood pressure values in 4th and 12th hours (98.0 ± 2.2 versus 94.5 ± 3.3 after 4th hour and 98.0 ± 2.2 versus 96.1 ± 3.2 after 12th hour). The respiratory rate following endotracheal extubation was significantly lower at the 4th hour in patients who have received HFO (*p* < 0.001); however, it did not differ significantly at the other follow-up intervals. Postoperative atelectasis score in the 4th hour was similar between groups (*p*=1.000); however, patients who have received HFO had significantly better atelectasis scores in 12th, 24th, 36th, and 48th hours (*p*=0.004, *p* < 0.001, *p* < 0.001, and*p* < 0.001, resp.). Preoperative FEV_1_ (forced expiratory volume) and FVC (forced vital capacity) were comparable between groups (*p*=0.650and*p*=0.228). However, during postoperative follow-up, HFO leads patients to achieve better FVC (*p* < 0.001) values. The dyspnea scores were significantly better in patients with HFO in both postoperative day 1 and day 2 (*p* < 0.001and*p* < 0.001). The comfort scores of patients who have received HFO were better in postoperative day 1 and day 2 (*p*=0.002and*p*=0.001, resp.). Postoperative parameters are summarized in Tables 3 and 4.

## 4. Discussion

Acute respiratory failure is still a challenging and life-threatening complication in patients who underwent open heart surgery with CPB. During respiratory insufficiency, respiratory support is a critical step to maintain patient comfort, prevent invasive mechanical ventilation, and decrease mortality [8]. Therefore, choosing the most appropriate device for oxygen therapy is a crucial decision to maintain adequate oxygenation especially in patients who are at high risk for acute respiratory failure such as patients with sleep apnea syndrome, advanced stage heart failure, and high BMI.

Obese patients are prone to hypoxia development due to several risk factors. Obese patients have extensive adipose tissue with high metabolic activity that is associated with increased oxygen consumption and carbon dioxide production. Due to breathing difficulties in obese cases, supportive muscles spend more oxygen and produce more carbon dioxide to overcome the workload. Additionally, compliance of respiratory organs and lung volumes are decreased in obese patients [9]. Due to the lower resting functional residual capacity, obese patients have an increased respiratory rate to compensate the ventilation-perfusion mismatch especially at the base of the lungs. Furthermore, postoperative atelectasis is common and more prominent in obese patients when compared with nonobese patients [10]. Thus, the best treatment modality to overcome postoperative oxygenation problems in obese patients is a critical problem and still under investigation.

The simple face mask and HFO nasal cannula are used for oxygen delivery in patients with hypoxia and/or hypercarbia. However, there is a trend in use of HFO especially in high-risk patients. Costello et al. stated that delivering oxygen through a face mask leads to dryness of the mouth and respiratory tract, and this situation leads to displacement of the face mask. Also, displacement of the mask is associated with the decrease in oxygen concentration [11]. On the other hand, HFO generates a dead space and creates a reservoir for oxygen in the respiratory system. In patients with tachypnea, delivered O_2_ may decrease due to the increased respiratory frequency, and HFO overcomes this by adequate and stable oxygenation. Moreover, HFO with positive pressure prevents the formation of atelectasis [12].

Previous reports on the efficiency of mask O_2_ and HFO in the management of acute respiratory failure had controversial results. Lemiale et al. investigated the effect of HFO in immunocompromised patients and found that HFO neither decreased the need for mechanical ventilation nor improved patients' quality of life [13]. In contrast, Schwabbauer et al. found significantly better dyspnea and patient comfort scores with HFO [12]. In another study, Maggiore et al. compared HFO and mask O_2_ in patients who were mechanically ventilated for more than 24 hours. They stated that high-flow O_2_ provided better oxygenation, fewer desaturation episodes, and lower reintubation rates [14]. Rittayamai et al. suggested HFO to overcome dyspnea and improve physiologic parameters [15]. In the present study, we achieved better PaO_2_, SpO_2_, and PCO_2_ levels in the first 48 hours with HFO in our obese patient cohort.

Pulmonary atelectasis is a common and undesired condition which is associated with oxygen impairment, decrease in lung compliance, increase in vascular resistance, and infectious complications [16]. To achieve more accurate classification of atelectasis and to evaluate treatment response, the Radiological Atelectasis Score was developed and validated. Parke et al. evaluated the effect of mask O_2_ and HFO by using Radiological Atelectasis Score, in 340 patients who underwent heart surgery. They claimed that prophylactic HFO improved the atelectasis scores [7]. Zarbock et al. analyzed 500 patients who underwent elective cardiac surgery and were supported by HFO to prevent atelectasis in the postoperative period [17]. In accordance with the literature, we have obtained better atelectasis scores in patients who received HFO. Thereby, we recommend HFO to prevent atelectasis in obese patients.

During oxygen therapy, patients' subjective response and patients' satisfaction have important roles in continuing oxygen treatment and mostly evaluated by scoring systems. Delclaux et al. analyzed patients who have received mask O_2_ treatment for acute hypoxemic respiratory insufficiency according to dyspnea scores. They found that applying O_2_ through a face mask had an impact on healing of dyspnea scores [18]. In another study by Stéphan et al., both mask O_2_ and HFO were found as beneficial tools to improve dyspnea scores to overcome hypoxia problem after cardiothoracic surgery. Also, Stéphan et al. emphasized that the two treatment modalities were not superior to each other according to dyspnea scores [6]. Kramer et al. demonstrated that 18% of mask O_2_ treatment failure was associated with mask discomfort [19]. Similarly, Calderini found mask discomfort as a reason for the cessation of treatment [20]. In our study, patients who received HFO had significantly better dyspnea and comfort scores. We analyzed only a specific group with BMI > 30 kg/m^2^, and this is emphasized to be the explanation of better dyspnea and comfort scores with HFO. Obese patients spend more effort on breathing and are more vulnerable to hypoxia. Thus, adequate oxygenation is mandatory to improve symptoms. Moreover, the treatment failure rate was higher with mask O_2_ and may have contributed to lower dyspnea and comfort scores in our patients.

Although the current study is a prospective randomized study in which we investigated the efficiency of HFO and compared HFO with conventional mask O_2_ in the postoperative period of obese patients who underwent open heart surgery, the research has certain limitations. The sample size in both groups in the study is modest. The cost of mask O_2_ and HFO treatments in obese patients was not investigated. Lastly, we have only evaluated the short-term results of both treatment modalities in statistically similar groups, and further researches with long-term follow-up outcomes may be helpful to strongly support the superiority of the technique to another.

In conclusion, we demonstrated that HFO therapy following CPB in obese patients improved postoperative PaO_2_, SpO_2_, and PaCO_2_ values and decreased atelectasis scores, reintubation, and mortality rates when compared with standard mask O_2_. Moreover, obese patients achieved significantly better dyspnea and comfort scores following HFO. Our findings could be supported by further prospective, randomized studies with larger patient volume.

## Figures and Tables

**Table 1:** Preoperative and operative characteristics of patients.

	Group		*p* value
	Mask O_2_ (*n* = 50)	High-flow O_2_ (*n* = 50)	
Sex (female/male)	32/18	32/18	1.000
Age (years)^∗^	61.3 ± 8.5	62.0 ± 6.7	0.660
Body mass index (kg/m^2^)^∗^	32.3 ± 1.1	32.5 ± 1.2	0.259
*Comorbidities*			
Diabetes mellitus	20	19	0.840
Hypertension	29	30	0.841
Chronic kidney disease	3	3	1.000
Smoking history	21	22	0.842
Ejection fraction (%)^∗^	51.3 ± 6.8	50.3 ± 6.2	0.445
Number of CPB^∗^	3.2 ± 0.7	3.2 ± 0.7	0.786
CPB duration (min)^∗^	90.5 ± 12.1	91.4 ± 13.0	0.709
Clamping duration (min)^∗^	41.0 ± 11.8	41.8 ± 12.8	0.740
Duration of extubation (hour)^∗^	8.2 ± 4.3	7.8 ± 1.4	0.529

^∗^Mean ± standard deviation; CPB: cardiopulmonary bypass; min: minutes.

**Table 2:** Postoperative complications, treatment failure, and mortality rates of patients.

	Group		*p* value
	Mask O_2_ (*n* = 50)	High-flow O_2_ (*n* = 50)	
CPAP requirement	11	6	0.187
Reintubation	4	0	**<0.001**
Pneumonia	2	0	**<0.001**
ICU stay (day)^∗^	2.8 ± 1.7	2.4 ± 0.5	0.130
Hospitalization duration (day)^∗^	6.9 ± 1.1	6.5 ± 0.7	**0.034**
Atrial fibrillation	12	7	**<0.001**
Transfusion rate^∗^	1.8 ± 0.7	2.0 ± 0.7	0.080
Mortality	2	0	**<0.001**

^∗^Mean ± standard deviation; CPAP: continuous positive airway pressure; ICU: intensive care unit.

**Table 3:** Postoperative respiratory characteristics, physical examination findings, and atelectasis scores of groups.

	Group		*p* value
	Mask O_2_ (*n* = 50)	High-flow O_2_ (*n* = 50)	
*Postoperative 4th hour* ^∗^			
PaO_2_	104.3 ± 5.6	112.3 ± 8.8	**<0.001**
SpO_2_	96.8 ± 1.3	98.0 ± 0.7	**<0.001**
PaCO_2_	43.1 ± 2.8	41.2 ± 2.1	**<0.001**
Heart rate (beats/min)	105.7 ± 8.0	98.4 ± 3.3	**<0.001**
Mean arterial pressure (mmHg)	94.5 ± 3.3	98.0 ± 2.2	**<0.001**
Respiratory rate (breaths/min)	21.6 ± 1.6	20.2 ± 0.8	**<0.001**
Atelectasis score	1 ± 0	1 ± 0	1.000
*Postoperative 12th hour* ^∗^			
PaO_2_	96.2 ± 7.4	106.9 ± 7.5	**<0.001**
SpO_2_	95.7 ± 1.4	97.5 ± 1.1	**<0.001**
PaCO_2_	43.7 ± 1.9	41.2 ± 2.1	**<0.001**
Heart rate (beats/min)	99.3 ± 6.1	96.4 ± 3.3	**0.009**
Mean arterial pressure (mmHg)	96.1 ± 3.2	98.0 ± 2.2	**0.001**
Respiratory rate (breaths/min)	20.1 ± 1.9	20.0 ± 0.9	0.845
Atelectasis score	1.7 ± 0.5	1.4 ± 0.5	**0.004**
*Postoperative 24th hour* ^∗^			
PaO_2_	96.6 ± 6.7	100.0 ± 4.5	**0.004**
SpO_2_	95.7 ± 1.3	97.4 ± 4.3	**0.001**
PaCO_2_	43.5 ± 1.8	40.7 ± 2.2	**<0.001**
Heart rate (beats/min)	100.7 ± 4.4	97.5 ± 4.9	**0.001**
Mean arterial pressure (mmHg)	96.6 ± 2.5	97.7 ± 6.5	0.277
Respiratory rate (breaths/min)	19.9 ± 1.7	19.9 ± 0.8	0.697
Atelectasis score	2.2 ± 0.5	1.7 ± 0.7	**<0.001**
*Postoperative 36th hour* ^∗^			
PaO_2_	97.1 ± 6.3	104.9 ± 5.9	**<0.001**
SpO_2_	95.8 ± 1.2	97.5 ± 1.2	**<0.001**
PaCO_2_	43.3 ± 1.6	39.3 ± 2.8	**<0.001**
Heart rate (beats/min)	100.6 ± 4.5	96.2 ± 4.3	**<0.001**
Mean arterial pressure (mmHg)	96.7 ± 2.5	95.4 ± 4.9	0.119
Respiratory rate (breaths/min)	19.6 ± 1.2	19.4 ± 0.9	0.322
Atelectasis score	2.2 ± 0.5	1.4 ± 0.5	**<0.001**
*Postoperative 48th hour* ^∗^			
PaO_2_	99.4 ± 7.1	106.0 ± 6.9	**<0.001**
SpO_2_	95.7 ± 1.3	97.5 ± 1.2	**<0.001**
PaCO_2_	42.3 ± 2.2	37.9 ± 2.6	**<0.001**
Heart rate (beats/min)	94.7 ± 4.6	95.1 ± 3.5	0.612
Mean arterial pressure (mmHg)	96.8 ± 2.4	97.0 ± 2.2	0.219
Respiratory rate (breaths/min)	19.5 ± 1.1	19.3 ± 0.9	0.451
Atelectasis score	1.7 ± 0.4	1.3 ± 0.5	**<0.001**

^∗^Mean ± standard deviation; PaO_2_: arterial pressure of oxygen; SpO_2_: peripheral oxygen saturation; PaCO_2_: arterial partial pressure of carbon dioxide; min: minutes.

**Table 4:** Comparison of spirometric parameters and dyspnea and comfort scores between groups.

	Group		*p* value
	Mask O_2_ (*n* = 50)	High-flow O_2_ (*n* = 50)	
Preoperative FEV_1_^∗^	2.1 ± 0.2	2.0 ± 0.2	0.650
Preoperative FVC^∗^	2.4 ± 0.5	2.5 ± 0.5	0.228
Postoperative FEV_1_^∗^	1.0 ± 0.2	1.1 ± 0.3	0.292
Postoperative FVC^∗^	1.1 ± 0.4	1.8 ± 0.4	**<0.001**
*Dyspnea score* (*1st day*)			
Improvement	19 (38%)	32 (64%)	**<0.001**
No improvement	20 (40%)	11 (22%)	—
Deterioration	11 (22%)	7 (14%)	—
*Dyspnea score* (*2nd day*)			
Improvement	27 (54%)	41 (82%)	**<0.001**
No improvement	17 (34%)	6 (12%)	—
Deterioration	6 (12%)	3 (6%)	—
*Comfort score* (*1st day*)	2.34 ± 1.65	3.79 ± 1.87	**0.002**
*Comfort score* (*2nd day*)	2.74 ± 1.45	4.05 ± 1.76	**0.001**

^∗^Mean ± standard deviation; FEV_1_: forced expiratory volume; FVC: forced vital capacity.

## Abbreviations

CPB: Cardiopulmonary bypass
HFO: High-flow O_2_
ICU: Intensive care unit
SBT: Spontaneous breathing trial
PEEP: Positive end-expiratory pressure
FiO_2_: Fraction of inspired oxygen
PaO_2_: Arterial pressure of oxygen
SpO_2_: Peripheral oxygen saturation
PaCO_2_: Arterial partial pressure of carbon dioxide
RAS: Radiological atelectasis score system
CPAP: Continuous positive airway pressure
FEV_1_: Forced expiratory volume
FVC: Forced vital capacity
Min: Minutes.
